# Association of p21 SNPs and risk of cervical cancer among Chinese women

**DOI:** 10.1186/1471-2407-12-589

**Published:** 2012-12-11

**Authors:** Ning Wang, Shizhuo Wang, Qiao Zhang, Yanming Lu, Heng Wei, Wei Li, Shulan Zhang, Duo Yin, Yangling Ou

**Affiliations:** 1 Department of Obstetrics and Gynecology, Shengjing Hospital of China Medical University, Shenyang, China

**Keywords:** P21, Single nucleotide polymorphism, Cervical cancer, Haplotype

## Abstract

**Background:**

The p21 codon 31 single nucleotide polymorphism (SNP), rs1801270, has been linked to cervical cancer but with controversial results. The aims of this study were to investigate the role of p21 SNP-rs1801270 and other untested p21 SNPs in the risk of cervical cancer in a Chinese population.

**Methods:**

We genotyped five p21 SNPs (rs762623, rs2395655, rs1801270, rs3176352, and rs1059234) using peripheral blood DNA from 393 cervical cancer patients and 434 controls.

**Results:**

The frequency of the rs1801270 A allele in patients (0.421) was significantly lower than that in controls (0.494, p = 0.003). The frequency of the rs3176352 C allele in cases (0.319) was significantly lower than that in controls (0.417, p < 0.001).The allele frequency of other three p21 SNPs showed not statistically significantly different between patients and controls. The rs1801270 AA genotype was associated with a decreased risk for the development of cervical cancer (OR = 0.583, 95%CI: 0.399 - 0.853, P = 0.005). We observed that the three p21 SNPs (rs1801270, rs3176352, and rs1059234) was in linkage disequilibrium (LD) and thus haplotype analysis was performed. The AGT haplotype (which includes the rs1801270A allele) was the most frequent haplotype among all subjects, and both homozygosity and heterozygosity for the AGT haplotype provided a protective effect from development of cervical cancer.

**Conclusions:**

We show an association between the p21 SNP rs1801270A allele and a decreased risk for cervical cancer in a population of Chinese women. The AGT haplotype formed by three p21 SNPs in LD (rs1801270, rs3176352 and rs1059234) also provided a protective effect in development of cervical cancer in this population.

## Background

Cervical cancer is the second most common cancer in women worldwide [1,2]. The human papilloma virus (HPV) appears to be a necessary factor in the development of almost all cases (> 90%) of cervical cancer [3]. The HPV E6 and E7 proteins are viral genes expressed in virtually all HPV-positive cervical carcinomas, and many experiments have shown that these are cooperative viral oncoproteins [4] that inactivate p53 and retinoblastoma (pRb) tumor suppressors, promoting carcinogenesis [4,5].

HPV infection is relatively common while only a small fraction of those infected develop cancer, suggesting that additional environmental, genetic, or immunological factors contribute to the progression to cervical cancer [6,7]. Cell cycle progression is regulated by cyclin-dependent kinases, crucial for normal growth and differentiation. Disruption of cell cycle control is common in cancer cells and is believed to play an essential role in cancer initiation and development. The cyclin-dependent kinase inhibitor p21 (also known as WAF1or CIP1) is encoded by the CDKN1A gene located on chromosome 6p21.2 [8,9]. The p21 protein binds to and inhibits the activity of cyclin-CDK2 or -CDK4 complexes, and disrupts cell cycle progression at G1 phase [10,11]. The expression of p21 is induced by the binding of tumor suppressor protein p53 to the p21 promoter [12-14]. The p21 protein can also interact with proliferating cell nuclear antigen (PCNA), a DNA polymerase accessory factor, and plays a regulatory role in S phase DNA replication and DNA damage repair [15]. Therefore, alteration in the p21 functional and/or promoter regions may adversely affect the regulation of cellular proliferation and increase susceptibility to cancer.

Identification of several genetic variants in p21 have been associated with cervical cancer [16,17]. The p21 single nucleotide polymorphism (SNP) rs1801270C/A, which occurs in codon 31, results in an amino acid substitution of arginine for serine. This polymorphism is located in a highly conserved region of p21 and is expected to affect its molecular function [18]. Prior studies have linked the p21 codon 31 SNP (rs1801270) to cervical cancer, with conflicting results [19,20]. In this study, we genotyped five p21 SNPs (rs1801270 at codon 31, rs762623 and rs2395655 in the promoter region, rs3176352 in an intron, and rs1059234 in the 3’ non-coding region) in 393 cervical cancer patients and 434 cancer-free controls to look for any associations between SNP alleles or genotypes and cervical cancer in a Chinese population.

## Methods

Before beginning this study, the study protocol was approved by the Ethics Committee of our hospital(Shengjing Hospital of China Medical University, Shenyang, China).

### Subjects

We ascertained 393 patients with cervical cancer between October, 2008 and September, 2011 at the Shengjing Hospital of China Medical University. Diagnosis of cervical cancer was confirmed by routine histopathological examination. A total of 434 control samples were randomly selected from the physical examination center from the same hospital and during the same period. The selection criteria for the controls included no individual history of cancer, no history of cervical intraepithelial neoplasia, normal cervical cytology and HPV examination, or normal pathological examination of the cervix within the past two years. HPV examination was performed as described previously [21]. At recruitment, written informed consent was obtained from all study subjects.

Age, smoking status, HPV status, and pathological stage of all 393 cervical cancer cases and 434 controls are presented in Table 1. The two groups seemed to be adequately matched in terms of age and smoking. Among the patients, 89.3% were HPV positive. The patients’ disease severity was assessed using the FIGO (1988) cervical cancer staging system (Table 1).

**Table 1:** Demographic and clinical data of the study group

	**Patients (n=393)**	**Controls (n=434)**	**P**^**a**^
	**No. (%)**	**No. (%)**	
Age			
≤35	82 (20.9)	105 (24.4)	0.377
36-50	219 (55.7)	241 (55.5)	
>50	92 (23.4)	88 (20.3)	
Smoking			
Never	371 (94.4)	419 (96.5)	0.177
Ever	22 (5.6)	15 (3.5)	
HPV status			
HPV+	351 (89.3)	0 (0)	
HPV-	42 (10.7)	434 (100)	
Clinical stage^b^			
Stage 0	213 (54.2)		
Stage I	138 (35.1)		
Stage II	34 (8.7)		
Stage III	7 (1.8)		
Stage IV	1 (0.3)		

### Genotype analysis

Genomic DNA was isolated from peripheral blood using the Tiangen Blood Genome Kit. Detailed primer and reaction information for the determination of the p21 SNP genotypes is given in Additional file 1: Table S1. Briefly, genotyping of rs762623 and rs2395655 was determined by DNA sequencing (Additional file 2: Figure S1). Genotyping of rs1801270, rs3176352, and rs1059234 was determined using a PCR-RFLP method. Each PCR reaction consisted of 0.1 mg DNA, 1 U Taq polymerase, 10 mM Tris–HCl (pH 8.3), 50 mM KCl, 1.0 mM MgCl_2_, 20 mM of each dNTP, and 0.2 μM of each primer. PCR products were digested with the indicated restriction enzymes and visualized on agarose gels (Additional file 3: Figure S2, Additional file 4: Figure S3, Additional file 5: Figure S4).

### Statistical analysis

Genotype and allele frequencies were used to assess conformity to Hardy–Weinberg equilibrium. Differences in age and smoking status between patients and controls were evaluated using the Chi-square test. The statistical effect of this study was calculated by Gpower 14 software (Faul & Erdfelder, Bonn, Germany). Post-hoc analysis (affect scale set to 0.2 and α set to 0.05) determined the statistical power of this study to be 0.86. Linkage disequilibrium (LD) between five loci of p21 was estimated by the likelihood-ratio test. A Bayesian statistical method was used to estimate the haplotypes for each subject based on their known genotypes. These analyses were implemented in Haploview software (version 4.2; Massachusetts Institute of Technology, Cambridge, MA). Haplotype construction and haplotype frequency analysis were performed by PHASE (version 2.1) software [22]. All other statistical analyses were performed using SPSS 13.0 software (SPSS Inc., Chicago, USA). After Bonferroni correction, differences were deemed significant at p < 0.01.

## Results

### Association of p21 SNP alleles and genotypes with cervical cancer risk

The frequencies of each allele of the five p21 SNPs (rs1801270, rs762623, rs2395655, rs3176352, and rs1059234) for patients and controls are given in Table 2. After performing Bonferroni correction to eliminate bias of multiple comparison, we found that the frequency of the rs1801270 A allele in cases (0.421) was statistically significantly lower than that found in controls (0.494; p = 0.003). Frequency of the rs3176352 C allele in cases (0.319) was also statistically significantly lower than that in controls (0.417; p < 0.001). The frequency of the rs1059234 alleles between cases and controls was close to statistical significance (p = 0.055). The frequencies of the other two polymorphisms were not statistically significant between the two groups (Table 2).

**Table 2:** Allele frequencies for all five p21 SNPs in cervical cancer patients and controls

	**Patient alleles**	**control alleles**		
	**(n = 786)**	**(n = 868)**		
**SNP**	**No. (%)**	**No. (%)**	**OR (95% CI)**	P*
rs762623				
A	151 (19.2)	167 (19.2)	1.001 (0.782-1.281)	0.993
G	635 (80.8)	701 (80.8)		
rs2395655				
A	399 (50.8)	406(46.8)	1.139 (0.938-1.383)	0.115
G	387 (49.2)	462 (53.2)		
rs1801270				
A	331 (42.1)	429 (49.4)	0.744 (0.613-0.904)	0.003
C	455 (57.8)	439 (50.6)		
rs3176352				
C	251(31.9)	317 (41.7)	0.656 (0.533-0.807)	0.000
G	535 (68.1)	551 (58.3)		
rs1059234				
C	422 (53.6)	425 (48.9)	1.208 (0.996-1.466)	0.055
T	364 (46.3)	443 (51.0)		

* P value was adjusted for age and smoking by multiple logistic regressions. After Bonferroni correction, significant P value was < 0.01.

The genotype distributions among both patients and controls (Table 3) were all in accordance with Hardy–Weinberg equilibrium. We used an unconditional logistic regression model adjusted for age and smoking to find any association between genotypes and the risk of cervical cancer (Table 3). We found that the rs1801270 AA genotype was associated with a decreased risk for the development of cervical cancer (OR = 0.583, 95% CI: 0.399 - 0.853, P = 0.005). This observation is consistent with the increased frequency of the rs1801270 A allele among controls. However, we did not observe a similar protective effect of the rs3176352 CC genotype even though the rs3176352 C allele frequency was lower in controls. No other genotypes of p21 SNPs were found to be associated with the risk of cervical cancer (Table 3).

**Table 3:** Genotype frequencies for five p21 SNPs in cervical cancer patients and controls

	**Patients**	**controls**		
	**(n=393)**	**(n=434)**		
**SNP**	**No. (%)**	**No. (%)**	**OR (95% CI)**	P*
rs762623				
AA	9 (2.3)	16 (3.7)	0.627 (0.272-1.447)	0.274
AG	133 (33.8)	135 (31.1)	1.096(0.816-1.472)	0.542
GG	251 (63.9)	283 (65.2)	reference	
rs2395655				
AA	96 (24.4)	92 (21.2)	1.393 (0.936-2.074)	0.102
AG	207 (52.7)	222 (51.2)	1.245 (0.892-1.738)	0.197
GG	90 (22.9)	120 (27.6)	reference	
rs1801270				
AA	76 (19.3)	117 (27.0)	0.583(0.399-0.853)	0.005
AC	179 (45.5)	195 (44.9)	0.811 (0.590-1.114)	0.196
CC	138 (35.1)	110 (28.1)	reference	
rs3176352				
CC	37 (9.4)	56 (12.9)	0.637 (0.339-1.016)	0.058
CG	177 (45.0)	205 (47.2)	0.883 (0.623-1.114)	0.218
GG	179 (45.5)	173 (39.9)	reference	
rs1059234				
CC	131 (33.3)	102 (23.5)	1.392 (0.957-2.023)	0.083
CT	160 (40.7)	221 (50.9)	0.786 (0.560-1.101)	0.161
TT	102 (26.0)	111 (25.6)	reference	

* P value was adjusted for age and smoking by multiple logistic regressions. After Bonferroni correction, significant P value was <0.01.

### Haplotype analysis of p21 SNPs

**Figure 1:**
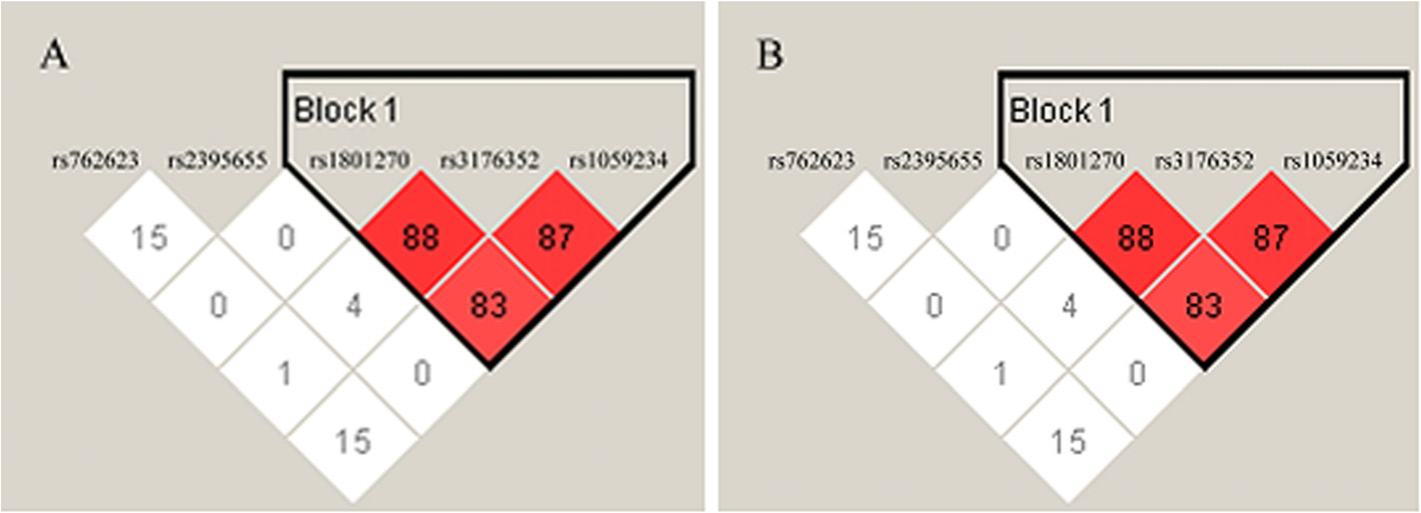
**Linkage disequilibrium (LD) plot and block structure of five p21 SNPs in 393 cervical cancer patients (A) and 434 cancer-free controls (B) showing LD of three SNPs.** In both groups, the haplotype block is based on confidence intervals D’. Each diamond represents the pairwise magnitude of LD, with red indicating strong LD (D’ > 0.8) and logarithm of odds score (LOD) ≥ 2.0.

Linkage disequilibrium (LD) analyses gave D’ values and r^2^ values (Additional file 6: Table S2), which suggested that three of the p21 SNP loci (rs1801270, rs3176352, and rs1059234) were in LD (Figure 1). The most common haplotypes were AGT, CCC, CGC, and CGT, accounting for 94.1% of the haplotypes observed in all individuals studied (Table 4). The frequency of AGT haplotype in cases (37.88%) was slightly lower than that in controls (44.42%), while the frequency of the CGC and CGT haplotypes were slightly higher in cases (20.92% and 7.07%, respectively) than in controls (11.63% and 3.91%, respectively; Table 4).

**Table 4:** Haplotypes of the three p21 SNPs (rs1801270, rs3176352, and rs1059234) in patients and controls

**Haplotype**		**Case (n=393)**	**Control (n=434)**
**ht**	**Haplotype**	**Percentage (%)(95% CI)**	**Percentage (%)(95% CI)**
1	AGT	37.88 (37.68-38.07)	44.42 (44.18-44.65)
2	CCC	29.30 (29.03-29.57)	33.11 (32.85-33.36)
3	CGC	20.92 (20.69-21.15)	11.63 (11.41-11.86)
4	CGT	7.07 (6.88-7.26)	3.91 (3.71-4.11)
5	AGC	2.20 (2.05-2.36)	3.52 (3.40-3.63)

Haplotypes with overall estimated frequency < 3% are not shown.

To determine any effect of a particular haplotype on the risk of cervical cancer, we looked at the frequency distribution of the four common p21 haplotypes among cases and controls. To further confirm the association between the p21 haplotype and the risk of cervical cancer, we selected the haplotypes of which the sum frequency in case and control is more than 5% and analyzed the association between the numbers of variants in each haplotype (ht1-AGT, ht2-CCC, ht3-CGC and ht4-CGT) and the risk of cervical cancer (Table 3). We found that homozygosity or heterozygostiy for the AGT haplotype provides a protective effect (Table 5). The OR value of the homozygous AGT haplotype is 0.643 (95% CI: 0.434 - 0.954, P = 0.028), while the OR value of the heterozygous AGT haplotype is 0.636 (95% CI: 0.467 - 0.865, P = 0.004). Homozygosity of the CGC haplotype significantly increased the risk of cervical cancer (OR=3.047; 95% CI: 1.788-5.192, P=0.000), heterozygostiy for the CGC haplotype had no effect on risk of cervical cancer (P = 0.321). Heterozygosity for the CGT haplotype was more common among patients, suggesting an increased risk of cervical cancer among CGT heterozygotes (OR = 1.846; 95% CI: 1.156 - 2.945, P = 0.010). Homozygosity for the CGT haplotype was not statistically significantly different among groups, possibly because of the small sample size (P=0.338). The frequency of the CCC haplotype was not statistically significantly different among the two groups and was therefore not associated with risk of cervical cancer in this population.

**Table 5:** Haplotypes of the three p21 SNPs in LD (rs1801270, rs3176352, and rs1059234) in patients and controls

	**Patients**	**controls**		
	**(n=393)**	**(n=434)**		
**haplotype**	**No. (%)**	**No. (%)**	**OR (95% CI)**	P*^a^
ht1-AGT				
**ht1/ht1**	**67 (17.0)**	**87 (20.0)**	**0.643 (0.434-0.954)**	**0.028**
**ht1/-**	**166 (42.2)**	**215(49.5)**	**0.636 (0.467-0.865)**	**0.004**
−/−	160(40.7)	132(30.4)	reference	
ht2- CCC				
ht2/ht2	26 (6.6)	41 (9.4)	0.628 (0.368-1.072)	0.088
ht2/-	183 (46.6)	210 (48.4)	0.865 (0.650-1.151)	0.321
−/−	184 (46.8)	183 (42.2)	reference	
ht3-CGC				
**ht3/ht3**	**51 (13.0)**	**21 (4.8)**	**3.047 (1.788-5.192)**	**0.000**
ht3/-	59 (15.0)	56 (12.9)	1.322 (0.888-1.970)	0.169
−/−	283 (72.0)	357 (82.3)	reference	
ht4- CGT				
ht4/ht4	3(0.8)	1(0.2)	3.054 (0.311-29.936)	0.338
**ht4/-**	**50 (12.7)**	**32 (7.4)**	**1.846 (1.156-2.945)**	**0.010**
−/−	340 (86.5)	401 (92.4)	reference	

* P value was adjusted for age and smoking by multiple logistic regressions.

## Discussion

This study looked for an association between five p21 SNPs (rs1801270, rs762623, rs2395655, rs3176352, rs1059234) and the risk of cervical cancer in a Chinese population. Studies have suggested an association between the p21 codon 31 polymorphism (rs1801270) and susceptibility to cancer, but with conflicting results [17,20,23,24]. Roh [17], Lee [23] and Jiang [24] found A allele seem to be a risk factor of cervical cancer, while Bhattacharya [20] and Tian [25] demonstrated C allele seem to be the risk factor. We found that the rs1801270 AA genotype and A allele occur more frequently in cancer-free controls, and seem to have a significant protective effect in Chinese women. Our results with the rs1801270 SNP are consistant with some previous reports [20,25], but differ from others [17,19,23,26], which found a deleterious effect of the rs1801270 A allele in cervical cancer. This discrepancy could be due to ethnic differences or sample size. Further, large-scale studies will be needed to validate the role of the p21 codon 31 SNP in cervical cancer.

Of the five p21 SNPs studied, three (rs1801270, rs3176352, and rs1059234) were found to be in linkage disequilibrium (LD). Haplotype analysis of these three p21 SNPs showed that haplotype AGT (which includes the rs1801270 A allele) occurs less frequently in cervical cancer patients than in controls. Our results suggest that both homozygosity and heterozygosity of the haplotype AGT may reduce the risk of cervical cancer in Chinese women. However, the heterozygosity of the haplotype CGT increases the risk of cervical cancer. In the current study, we found the association of the other two loci (rs3176352 and rs1059234) and cervical cancer tended to statistical significance. However, the role of the two p21 polymorphisms needs to be studied in a larger cervical cancer patient cohort in the future.

The molecular mechanism underlying the protective effect of the rs1801270 A allele in cervical cancer is unclear. This C/A SNP within the p21 codon 31 results in a substitution of arginine for serine in a conserved region of the protein and may affect the DNA binding affinity and thus physiological function of p21 [27]. The p21 protein plays a role in cell cycle regulation. It binds to cyclin-CDK complexes to arrest cell cycle progression at G1 phase. The transcription of p21 is partially regulated by p53, which binds to the p21 promoter and induces expression of p21. Therefore, functional domains as well as the promoter region of p21 could be affected by SNPs and lead to altered p21 functionality [26]. The rs2395655 SNP is located in the transcription factor E2F binding region. The rs1059234 SNP occurs in the 3’-non coding region and may affect p21 mRNA stability. Although our results do not find an association between these SNPs and cervical cancer, the molecular mechanism(s) through which p21 SNPs affect risk of cervical cancer is a field that should be pursued further.

Studies of p21 expression in cervical cancer have reported higher levels of expression of p21 in cancer tissue compared to normal tissue [28,29], while others showed there is no association between the expression of p21 and the pathological features of cervical cancer [30]. After infection with HPV, the E6 protein of HPV promotes p53 degradation and thus inhibits p21 expression and function. Loss of p21 results in decreased ability of the cell to stop cell cycle progression, possibly leading to carcinogenesis. However, the molecular mechanisms that mediate carcinogenesis are very complex, and this is not the only plausible explanation of the relationship between p21, p53, HPV, and cancer.

## Conclusion

In conclusion, our study showed a significant association between the A allele of the p21 rs1801270 SNP and decreased risk of cervical cancer in a Chinese population. Furthermore, a haplotype containing the rs1801270 A allele was also associated with decreased risk of cervical cancer in this population. The molecular mechanisms underlying this association remain to be determined.

## Abbreviations

HPV: Human Papillomavirus; SNP: Single nucleotide polymorphism; HWE: Hardy–Weinberg Equilibrium; MAF: Minor allele frequency; PCR-RFLP: Polymerase chain reaction-restriction fragment length polymorphism; LD: Linkage disequilibrium; FIGO: International Federation of Gynecology and Obstetrics.

## Competing interests

The authors declare no conflict of interest.

## Authors’ contributions

NW participated in the design of the study and sequencing. SW contributed to PCR-RFLP. QZ carried out sample collection and management. YL participated in statistical analysis. HW carried out DNA extraction. WL participated in sequencing. SZ participated in the design of the study. YD participated in statistical analysis. OY contributed to PCR-RFLP. All authors read and approved the final manuscript.

## Pre-publication history

The pre-publication history for this paper can be accessed here:

http://www.biomedcentral.com/1471-2407/12/589/prepub

## Supplementary Material

Additional file 1 **Table S1.** Methods used for p21 SNP genotyping. Click here for file

Additional file 2 **Figure S1.** Sample sequence tracing of rs762623 and rs2395655 SNPs showing homozygosity for each allele (top and bottom) or heterozygosity (middle). Click here for file

Additional file 3 **Figure S2.** PCR-RFLP analysis of p21 SNP rs1801270A/C. The 310 bp PCR products were digested by BglI, and presence of the C allele resulted in two bands of sizes 239 and 71 bp. Electrophoresis was performed in 3% agarose gel. Click here for file

Additional file 4 **Figure 3.** PCR-RFLP analysis of p21 SNP rs3176352A/C. The 448 bp PCR products were digested by ApaLI, and presence of the C allele resulted in two bands of sizes 289 and 159 bp. Electrophoresis was performed in 2.5% agarose gel. Click here for file

Additional file 5 **Figure S4.** PCR-RFLP analysis of p21 SNP rs1059234T/C. The 480 bp PCR products were digested by PstI, and presence of the C allele resulted in two bands of sizes 291 and 189 bp. Electrophoresis was performed in 2.5% agarose gel. (TIFF 571 kb) Click here for file

Additional file 6 **Table S2.** Pairwise linkage disequilibrium analysis for all five p21 SNPs for all subjects studied. Click here for file
