# Supplementary material for: Association of p21 SNPs and risk of cervical cancer among Chinese women
Source: BMC Cancer. 2012 Dec 11;12:589. doi: 10.1186/1471-2407-12-589 (PMC3527144; doi:10.1186/1471-2407-12-589)
Supplement: Additional file 1 — Table S1. Methods used for p21 SNP genotyping. [file 1471-2407-12-589-S1.doc]

| SNP | Location | Method | Primers | Enzyme | MAF | HWE(p) |
| --- | --- | --- | --- | --- | --- | --- |
| rs762623 | 36645466 | sequencing | F: CTCCAAGCCTGGGTTCTGT R: TCTGGCAGGCAAGGATTTAC |  | 0.192 | 0.261 |
| rs2395655 | 36645696 | sequencing | F: CTCCAAGCCTGGGTTCTGT R: TCTGGCAGGCAAGGATTTAC |  | 0.487 | 0.311 |
| rs1801270 | 36651971 | PCR-RFLP | F: ATGTCCGTCAGAACCCAT R: TGGTCTTCCTCTGCTGTC | BlpI | 0.459 | 0.012 |
| rs3176352 | 36652339 | PCR-RFLP | F: GACACCACTGGAGGGTGACT R: GGTCTTTGCTGCCTACTTGC | ApaL I | 0.343 | 0.547 |
| rs1059234 | 36653597 | PCR-RFLP | F:TGCGGTGATGGATAAAATCA R:CATTCAACCGCCTAGTTTTTG | PstI | 0.488 | 0.028 |

SNP rs numbers and locations are according to the dbSNP database www.ncbi.nlm.gov/projects/SNP. MAF: minor allele frequency ; HWE: Hardy–Weinberg equilibrium.
