# Supplementary figures and images for: Association of p21 SNPs and risk of cervical cancer among Chinese women
Source: BMC Cancer. 2012 Dec 11;12:589. doi: 10.1186/1471-2407-12-589 (PMC3527144; doi:10.1186/1471-2407-12-589)

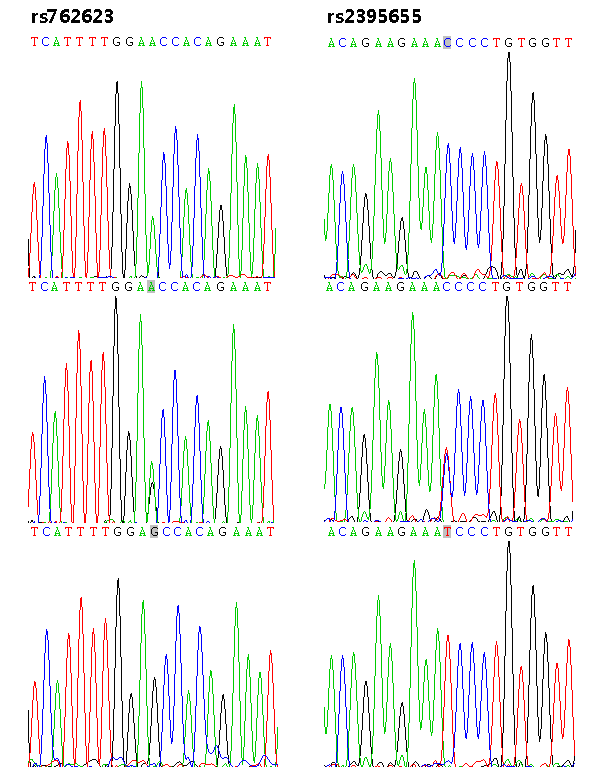

Supplement: Additional file 2 — Figure S1. Sample sequence tracing of rs762623 and rs2395655 SNPs showing homozygosity for each allele (top and bottom) or heterozygosity (middle). [file 1471-2407-12-589-S2.tiff]

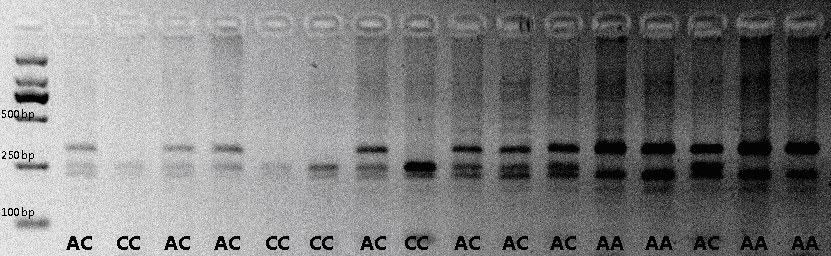

Supplement: Additional file 3 — Figure S2. PCR-RFLP analysis of p21 SNP rs1801270A/C. The 310 bp PCR products were digested by BglI, and presence of the C allele resulted in two bands of sizes 239 and 71 bp. Electrophoresis was performed in 3% agarose gel. [file 1471-2407-12-589-S3.tiff]

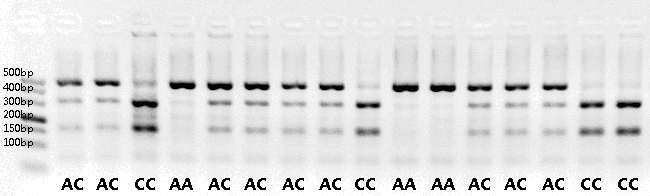

Supplement: Additional file 4 — Figure 3. PCR-RFLP analysis of p21 SNP rs3176352A/C. The 448 bp PCR products were digested by ApaLI, and presence of the C allele resulted in two bands of sizes 289 and 159 bp. Electrophoresis was performed in 2.5% agarose gel. [file 1471-2407-12-589-S4.tiff]

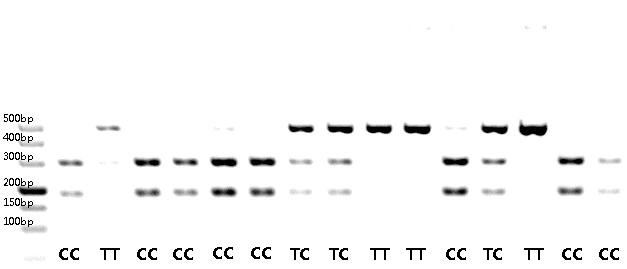

Supplement: Additional file 5 — Figure S4. PCR-RFLP analysis of p21 SNP rs1059234T/C. The 480 bp PCR products were digested by PstI, and presence of the C allele resulted in two bands of sizes 291 and 189 bp. Electrophoresis was performed in 2.5% agarose gel. (TIFF 571 kb) [file 1471-2407-12-589-S5.tiff]
