# Supplementary material for: Association of p21 SNPs and risk of cervical cancer among Chinese women
Source: BMC Cancer. 2012 Dec 11;12:589. doi: 10.1186/1471-2407-12-589 (PMC3527144; doi:10.1186/1471-2407-12-589)
Supplement: Additional file 6 — Table S2. Pairwise linkage disequilibrium analysis for all five p21 SNPs for all subjects studied. [file 1471-2407-12-589-S6.doc]

| SNP Name | rs762623 | rs2395655 | rs1801270 | rs3176352 | rs1059234 |
| --- | --- | --- | --- | --- | --- |
| rs762623 | -- | 0.004 | 0.0 | 0.0 | 0.003 |
| rs2395655 | 0.136 | -- | 0.0 | 0.001 | 0.001 |
| rs1801270 | 0.016 | 0.019 | -- | 0.346 | 0.619 |
| rs3176352 | 0.034 | 0.046 | 0.882 | -- | 0.378 |
| rs1059234 | 0.111 | 0.026 | 0.833 | 0.871 | -- |

D’ values are given in the lower left, r^2^ values are given in the upper right; D’>0.8 and r^2^>0.3 are considered significant linkage disequilibrium.
